# pH-regulated template-free assembly of Sb_4_O_5_Cl_2_ hollow microsphere crystallites with self-narrowed bandgap and optimized photocatalytic performance

**DOI:** 10.1038/srep27765

**Published:** 2016-06-16

**Authors:** Liuqing Yang, Jianfeng Huang, Liyun Cao, Li Shi, Qing Yu, Xingang Kong, Yanni Jie

**Affiliations:** 1 School of Materials Science and Engineering, Shaanxi University of Science and Technology, Weiyang, Xi’an, Shaanxi 710021, P.R. China; 2 Environmental Remediation Materials Unit, National Institute for Materials Science (NIMS), 1-1 Namiki, Tsukuba, Ibaraki 305-0044, Japan

## Abstract

Sb_4_O_5_Cl_2_ hollow microspheres with self-narrowed bandgap and optimized photocatalytic performances are synthesized via a facile template-free method. It is found that the crystal structure and morphology of Sb_4_O_5_Cl_2_ crystallites are strongly dependent on the pH values of precursors. Nano-sized irregular-cuboids assembled Sb_4_O_5_Cl_2_ micro-particles and hollow microspheres can be synthesized at pH 1 and 2, whereas individual Sb_4_O_5_Cl_2_ micro-belts become to form when the pH is higher than 3. The irregular-cuboids assembled Sb_4_O_5_Cl_2_ micro-particles and hollow microspheres exhibit self-narrowed bandgap and higher light absorption ability compared with individual Sb_4_O_5_Cl_2_ micro-belts. The photoelectrochemical measurements show that the assembled Sb_4_O_5_Cl_2_ hollow microsphere crystallites prepared at pH 2 exhibit enhanced carrier density, improved separation efficiency of electron-hole pairs and decreased electron-transfer resistance. As a result, the irregular-cuboids assembled Sb_4_O_5_Cl_2_ hollow microspheres prepared at pH = 2 exhibit the highest photocatalytic activity for the degradation of gaseous iso-propanol (IPA) and Rhodamine B (RhB) aqueous solution. The good photocatalytic activity of Sb_4_O_5_Cl_2_ sample prepared at pH = 2 may be caused by the synergistic effect of its higher light absorption, the decreased electron-transfer resistance, the suppressed recombination of photogenerated electrons and holes, and the increased surface area.

With the shortage of energy and the increasingly serious problem of environmental pollution, photocatalysis which owns the outstanding characteristic of low cost and environmental friendliness, has attracted intensive attention in view of converting light energy into hydrogen or oxygen^1^,^2^, photocatalytic reduction of CO_2_^3^, and degrading organic contaminants into inorganic substance^4^,^5^. The exploration of photocatalytic materials with well-controlled morphology and effective tuning of light absorption and bandgaps for the corresponding photocatalytic applications have attracted wide interest over the past decade. As a result, a great deal of efforts have been taken on the design of semiconductor materials with precisely tailored bandgaps which are strongly dependent not only on their composition^6^, but also on the structure^7^, phase^8^, size, shape^9^ and surface functionalization^10^ for better photocatalytic properties.

Narrowing bandgap of semiconductors, which can permit valence electrons to be excited by the photons with lower energy and thus harvest a larger portion of solar radiation, has been regarded as an effective strategy and extensively pursued to efficiently utilize solar energy within semiconductors to achieve better photocatalytic activities^11^. Among various strategies to narrow the bandgap of semiconductors, impurity doping has been widely adopted^12^,^13^,^14^,^15^. However, doping strategy is not ideal for all purposes and the introduction of impurity states incurs the risk of suppressing photo-electronic conversion efficiency^16^. In addition, doping operation usually requires complex preparation process and special conditions, which is difficult to be realized in some cases^17^. As another effective strategy to narrow the bandgap of semiconductors, the electronic coupling strategy has also been adopted and it has been successfully realized in CdS nanostructures^18^ and TiO_2_ nanoparticles assemblies^19^. The electronic coupling of interfacial cation-cation pairs can bring about not only self-narrowed bandgap but also some desirable and novel properties to the assembly of semiconductors nanoparticles compared with individual particles as well as bulk materials. Consequently, the semiconductor nanoparticles assemblies could achieve superior aptitude for solar energy utilization.

Antimony oxychloride has drawn considerable interest because of their applications in energy-storage materials, catalyst and flame retardant. Among various antimony oxychloride materials, Sb_4_O_5_Cl_2_ has been extensively studied, because it can greatly reduce the dosage of the coloring matter and improve the transparency of plastic products compared with other antimony family flame retardants, and it also exhibits excellent flame retardant properties when used alone or in conjunction with a halogenated organic compound^20^,^21^,^22^. As a semiconductor, Sb_4_O_5_Cl_2_ has also been coupled with other photocatalyts to improve the photocatalytic performance for degradation of methyl orange^23^,^24^. Sb_4_O_5_Cl_2_ crystallites with various morphologies such as nanobelts, nanowires, nanowire-flower, bundles of nanowires, bundles of flakes and hollow prisms have been obtained by solution and hydrothermal methods^22^,^25^,^26^. However, to the best of our knowledge, there are almost no reports about the preparation of Sb_4_O_5_Cl_2_ hollow spheres through a facile template-free solution route. Fabricating the hollow sphere structures through a simple template-free method is of great value, since the addition of the templates to the reaction systems would complicate the synthetic procedures and also may introduce some uncertain impurities that may be disadvantageous to the properties of the final products. Moreover, although some works have been done on Sb_4_O_5_Cl_2_, rarely has been reported on its electronic structure, photoelectrochemical properties and photocatalytic performance.

In this study, we report a facile template-free method for the synthesis of hollow microspheres assembled by Sb_4_O_5_Cl_2_ nanoparticles. Such architecture is fabricated by just adjusting the pH value of precursor, showing self-narrowed bandgap and enhanced photocatalytic performance compared with the individual Sb_4_O_5_Cl_2_ crystallites. More specifically, irregular-cuboids assembled Sb_4_O_5_Cl_2_ micro-particles and hollow microspheres can be synthesized at pH 1 and 2, whereas individual Sb_4_O_5_Cl_2_ micro-belts became to form when the pH is higher than 3. The inter-particle electronic coupling provides the irregular-cuboids assembled Sb_4_O_5_Cl_2_ crystallites with self-narrowed bandgap compared with that of individual Sb_4_O_5_Cl_2_ micro-belts. It was found that the Sb_4_O_5_Cl_2_ hollow microspheres assembled by irregular-cuboids prepared at pH = 2 showed a higher photocatalytic activity than the other four samples for photocatalytic degradation of gaseous iso-propanol (IPA) and Rhodamine B (RhB) aqueous solution, which was ascribed to the synergistic effect of its higher light absorption, the decreased electron-transfer resistance, the suppressed recombination of photogenerated electrons and holes, and the increased surface area.

## Results

**Figure 1:**
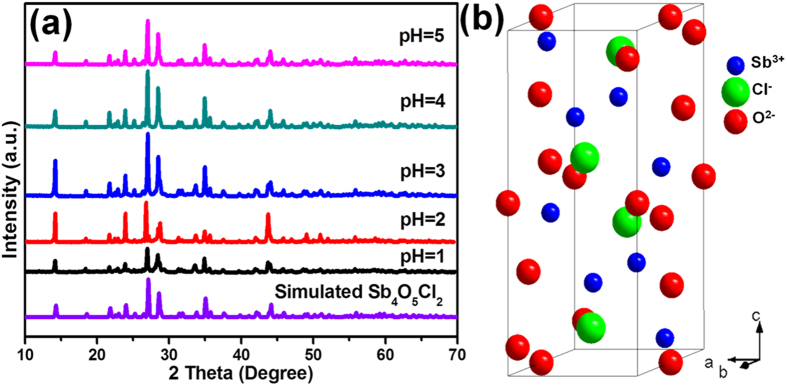
(**a**) XRD patterns of the samples prepared at precursor pH = 1, 2, 3, 4 and 5 through hydrothermal process (T = 160 °C, t = 12 h) and (**b**) a perfect unit cell of monoclinic Sb_4_O_5_Cl_2_ structure.

The Sb_4_O_5_Cl_2_ powders used in this study were prepared from a one-step hydrothermal process (See Methods). The crystallographic structure of the products was revealed by X-ray diffraction (XRD) measurement. As shown in Fig. 1(a), the samples prepared at pH values ranging from 1 to 5 exhibit similar diffraction patterns and all the diffraction peaks in each sample can be indexed to monoclinic Sb_4_O_5_Cl_2_ structure^20^ and no peaks for other phases are observed, which demonstrates the high purity of phase structure of the as-prepared Sb_4_O_5_Cl_2_. The unit cell of monoclinic Sb_4_O_5_Cl_2_ structure (space group: P21/c, PDF-number: 01-070-1102) is shown in Fig. 1(b). Sb_2_O_3_ which is consistent with previous report^21^ began to form when the precursor pH value was adjusted to 6 (Supplementary Fig. S1). The above information shows that the initial pH value of the reactant plays a momentous role in the synthesis of Sb_4_O_5_Cl_2_ crystallites, which is consistent with previously reported results^22^,^25^,^26^ that the OH^−^ have an important effect on the hydrolysis process. In this work, OH^−^ are supplied by ammonia. In the presence of OH^−^, the reaction can be shown by the following equations^27^,^28^,^29^,^30^:


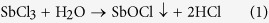



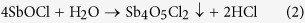



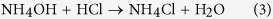



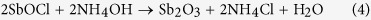



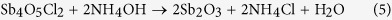


According to the above equations, it shows that the excess addition of NH_4_OH would produce Sb_2_O_3_ from Sb_4_O_5_Cl_2_, and this can explain the appearance of the Sb_2_O_3_ phase when the precursor pH was adjusted to 6 by adding more ammonia solution.

**Figure 2:**
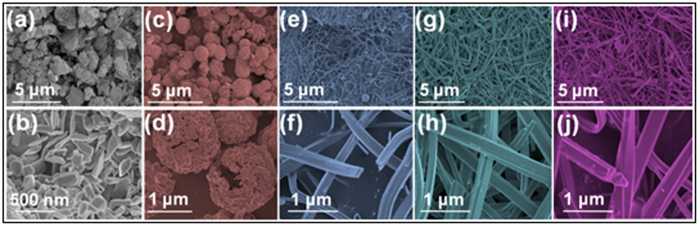
SEM images of the Sb_4_O_5_Cl_2_ samples prepared at pH values of (**a,b**) 1, (**c,d**) 2, (**e,f**) 3, (**g,h**) 4 and (**i,j**) 5 through hydrothermal process (T = 160 °C, t = 12 h).

Figure 2(a–j) display the scanning electron microscopy (SEM) and the magnified SEM images of the Sb_4_O_5_Cl_2_ samples obtained from hydrothermal process at 160 °C for 12 h at pH = 1, 2, 3, 4 and 5, respectively. It is obvious that the pH value of the precursor solution had a crucial influence on the morphology of Sb_4_O_5_Cl_2_ crystallites. The panoramic SEM image of the sample obtained at pH = 1 in Fig. 2(a) illustrates the irregular micro-particles in various micro-sizes. The corresponding magnified SEM image in Fig. 2(b) presents that the large micro-particles of sample is assembled by large amount of irregular-cuboids (about 200 nm in length, 50 nm in width and 20 nm in thickness). Figure 2(c) shows that the Sb_4_O_5_Cl_2_ sample prepared at pH = 2 appears as the spheres morphology whose diameters are distributed from hundreds of nanometers to several micrometers. The corresponding magnified image in Fig. 2(d) shows that the sphere is hollow and assembled by several nano-sized irregular-cuboids. However, the Sb_4_O_5_Cl_2_ crystallites with belts morphology began to appear when the pH value is adjusted to 3, and the length of the belts is up to tens of micrometers, as shown in Fig. 2(e,f). Then at pH = 4 and 5, as shown in Fig. 2(g–j), individual Sb_4_O_5_Cl_2_ crystallites with belts-like structures are also obtained. The SEM observation indicates that the Sb_4_O_5_Cl_2_ crystallites prepared at pH = 1 and 2 show the assembled nanostructure, where as individual Sb_4_O_5_Cl_2_ micro-belts became to form when the precursor pH values are higher than 3. The assembled Sb_4_O_5_Cl_2_ structures prepared at pH = 1 and 2 would show novel and useful collective properties in electronic structure and catalysis compared with those of the individual Sb_4_O_5_Cl_2_ crystallites prepared at pH = 3, 4 and 5, due to the inter-particle electronic coupling mechanism, and this will be discussed in the following part in detail. Similar phenomena were also observed in a previous work^18^,^19^, in which nanoparticle assemblies yielded much narrower bandgaps, red-shifted optical absorption bands and higher photocatalytic activities compared with those of individual particle materials.

**Figure 3:**
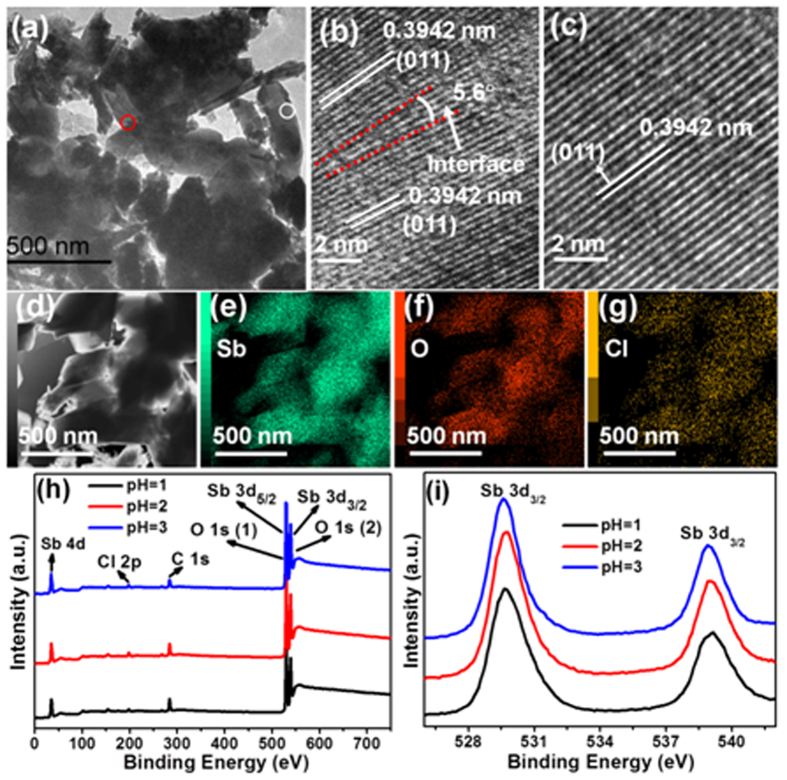
(**a**) TEM image of part of a sphere assembled by Sb_4_O_5_Cl_2_ particles prepared at pH = 2, (**b**) HRTEM image of an interface between two adjacent particles captured from the red circle in (**a**), and (**c**) HRTEM image of one particle captured from the white circle in (**a**). (**d**) TEM image of assembled Sb_4_O_5_Cl_2_ particles prepared at pH = 2, and (**e–g**) the corresponding elemental mapping images of Sb, O and Cl, respectively. (**h**) XPS spectra of the as-prepared Sb_4_O_5_Cl_2_ samples prepared at pH = 1, 2 and 3, and (**i**) high resolution XPS spectra of Sb 3d.

Transmission electron microscopy (TEM) and high-resolution TEM characterization on Sb_4_O_5_Cl_2_ sample prepared at pH = 2 in Fig. 3(a–c) are carried out to investigate the nanoparticle assemblies. Figure 3(a) clearly shows that the Sb_4_O_5_Cl_2_ crystallites are assembled by several nano-sized irregular-cuboids. Figure 3(b) presents the HRTEM image of an interface between two adjacent Sb_4_O_5_Cl_2_ irregular-cuboids captured from the red circle in Fig. 3(a). It shows that the interface is parallel to the (011) plane at one irregular-cuboid side and contains an included angle of 5.6° against the (011) plane at another irregular-cuboid side. Slight dislocation is observed at the interface, showing coherent lattice combination between the two irregular-cuboids. The HRTEM image of an individual irregular-cuboid is also taken and shown in Fig. 3(c). The well-resolved lattice fringe with interplanar distances of 0.3942 nm is indexed to the (011) planes of Sb_4_O_5_Cl_2_, and no dislocation is observed. The TEM image and its corresponding elemental mapping images in Fig. 3(d–g) show that the elements Sb, O, Cl are homogeneously distributed in the assembled Sb_4_O_5_Cl_2_ particles, demonstrating the good homogeneity of the composition of the particles. The TEM images show that at the interface of two adjacent Sb_4_O_5_Cl_2_ particles, the outer atoms are in contact, which will induce electronic coupling between these Sb_4_O_5_Cl_2_ particle assemblies^18^,^19^. This electronic coupling has been further detected by X-ray photoelectron spectroscopy (XPS) measurement.

Previous research reported that when two particles are electronically coupled, bonding and anti-bonding levels are formed, yielding a new electronic structure^18^,^19^. In order to reveal the electron coupling phenomenon in the assembled Sb_4_O_5_Cl_2_ crystallites prepared at pH = 1 and 2, the samples prepared at pH = 1, 2 and 3 were selected as typical samples to perform XPS measurements, as the samples prepared at pH 4 and 5 contain similar individual micro-belts morphology as that of the samples prepared at pH 3. Figure 3(h) illustrates the existence of the Sb, Cl, O and C elements in the three samples^22^. The binding energies of 33 and 200 eV belong to Sb 4d and Cl 2p respectively, indicating that the oxidation states of Sb and Cl are +3 and −1^31^. The peak marked with O1s belongs to O atoms coming from the samples and the gas (such as CO_2_) absorbed in the surface of the samples and the peak marked with C1s in the spectrum is attributed to the C element of CO_2_^32^. Figure 3(i) presents the high-resolution XPS spectra of Sb 3d for a better identification of Sb chemical state. The characteristic peaks at around 529 eV and 539 eV are ascribed to Sb 3d_5/2_ and Sb 3d_3/2_, respectively and no peak of Sb(V) is observed at 540.3 eV, which indicates that Sb in as-prepared Sb_4_O_5_Cl_2_ samples exists only in Sb(III) state^23^. The doublet peaks attributed to Sb 3d of Sb_4_O_5_Cl_2_ sample prepared at pH = 1 and 2 show similar binding energy. However, when compared with the individual Sb_4_O_5_Cl_2_ micro-belts prepared at pH = 3, the Sb_4_O_5_Cl_2_ assemblies prepared pH = 1 and 2 show an increase of 0.1 eV in the binding energy of Sb electrons. This binding energy increase suggests that the outer Sb atoms might be in contact in the inter-particles, and demonstrates that the inter-particle electronic coupling in the Sb_4_O_5_Cl_2_ assemblies prepared at pH = 1 and 2 is realized. We also compare the XPS spectra of particle-assembled sample prepared at pH = 2 with individual particle sample that have similar particle size to investigate the inter-particle electronic coupling. As shown in Supplementary Fig. S2, when compared with the individual Sb_4_O_5_Cl_2_ particle sample, the Sb_4_O_5_Cl_2_ particle-assembled sample also shows an increase of 0.1 eV in the binding energy of Sb electrons. This binding energy increase further proves that the inter-particle electronic coupling exists in the Sb_4_O_5_Cl_2_ assemblies.

**Figure 4:**
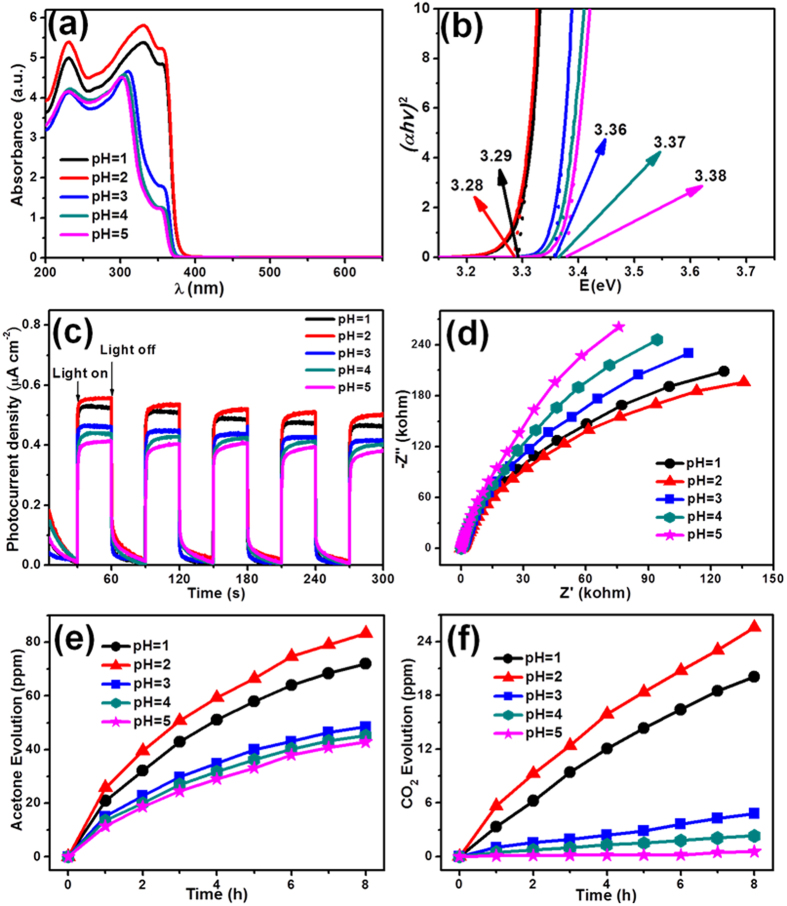
(**a**) UV–vis diffuse reflectance spectra, (**b**) bandgap energies, (**c**) the photocurrent density, and (**d**) AC impedance image of the Sb_4_O_5_Cl_2_ samples prepared at pH = 1, 2, 3, 4 and 5. (**e**) Photocatalytic acetone evolution and (**f**) CO_2_ evolution curves over the Sb_4_O_5_Cl_2_ samples prepared at pH = 1, 2, 3, 4 and 5 for photocatalytic IPA degradation, respectively.

The electronic band structure of the resulting Sb_4_O_5_Cl_2_ crystallite samples was investigated by a combined analysis of UV-visible absorption spectra and the Mott-Schottky plots. The absorption spectra of the samples are given in Fig. 4(a). It is obvious that these five samples can only adsorb the UV light shorter than 380 nm and the Sb_4_O_5_Cl_2_ sample prepared at pH = 2 possess the best ability to absorb UV light. The absorption edge is 379 nm for Sb_4_O_5_Cl_2_ sample prepared at pH = 1. And the absorption edge of Sb_4_O_5_Cl_2_ sample prepared at pH = 2 is similar to that of the sample prepared at pH = 1. The absorption edges of Sb_4_O_5_Cl_2_ sample prepared at pH = 3, 4 and 5 also show similar positions, and all have a obvious blue shift when comparing with the samples prepared at pH = 1 and are 371, 370 and 368 nm, respectively. As a conventional semiconductor, the direct band gap energies of the samples can be calculated by the equation (αhν)^2^ = A(hν-Eg), where α, ν, A and Eg are absorption coefficient, light frequency, proportionality constant and band gap energy, respectively^33^. The band gap energies of the Sb_4_O_5_Cl_2_ samples prepared at pH = 1, 2, 3, 4 and 5 are estimated to be 3.29, 3.28, 3.36, 3.37 and 3.38 eV, respectively, calculated from the intercept of the tangents to the plots shown in Fig. 4(b). It is apparent that the samples prepared at pH = 1 and 2 present similar bandgap, which are much narrower compared with those of the samples prepared at 3, 4 and 5. This means that the self-narrowed bandgap can be realized in Sb_4_O_5_Cl_2_ crystallites through simply adjusting the pH values of the precursors. The bandgap self-narrowing phenomenon can be explained by interfacial electronic coupling between adjacent Sb_4_O_5_Cl_2_ crystallites at pH = 1 and 2, as evidenced by the SEM, TEM observations and XPS measurements.

The flat-band potential of Sb_4_O_5_Cl_2_ crystallites is measured using the electrochemical method and the Mott-Schottky plots are shown in Supplementary Fig. S3. The Mott-Schottky plots of the Sb_4_O_5_Cl_2_ electrode apparently disclose the typical n-type characteristic inorganic semiconductors owing to the positive slope of the linear plots^34^. E_FB_ is strongly related to the bottom of the E_CB_ and is considered to be located just under the E_CB_ for n-type semiconductors^35^,^36^. According to the smallest band gap of Sb_4_O_5_Cl_2_ prepared at pH = 2, the top of E_VB_ of Sb_4_O_5_Cl_2_ prepared at pH = 2 is calculated to be −0.25 eV, which is more negative than those of Sb_4_O_5_Cl_2_ prepared at pH = 1, 3, 4 and 5 (−0.20, −0.18, −0.13 and −0.10 eV, respectively). The carrier density can be estimated by the slope of the Mott-Schottky plot and smaller slopes of Mott–Schottky plots reveal the enhanced carrier densities^15^,^37^. Thus among the five samples, Sb_4_O_5_Cl_2_ assemblies prepared at pH = 2, which has the smallest slope, has the highest carrier densities.

The photoelectrochemical properties of the Sb_4_O_5_Cl_2_ crystallites were studied by measuring the photocurrent response of Sb_4_O_5_Cl_2_ crystallites photoanode. As shown in Fig. 4(c), the Sb_4_O_5_Cl_2_ crystallites photoanodes in a photoelectrochemical cell with a Na_2_SO_4_ solution as the electrolyte gave an apparent response to light on/off switching cycles at the bias applied at 0.5 V under simulated solar light irradiation. Sb_4_O_5_Cl_2_ crystallites prepared at different precursor pH values show different photocurrent density because of the different separation rate of the photogenerated electrons and holes^38^. With the increase of pH values, the photocurrrent density of the samples is increased firstly, reach the highest value at pH = 2 then decrease. It indicates the best separation of electron-hole pairs in Sb_4_O_5_Cl_2_ crystallites prepared at pH = 2. The electrochemical impedance spectroscopy in Fig. 4(d) indicates the decreased electron-transfer resistance in Sb_4_O_5_Cl_2_ crystallites prepared at pH = 2, because of the smallest diameter of the semicircular Nyquist plots^39^. Combining the results of the transient photocurrent and electrochemical impedance measurements, it is clear that the separation efficiency of the photoinduced electron-hole pairs is improved in Sb_4_O_5_Cl_2_ crystallites prepared pH = 2, which would result in a higher photocatalytic activity.

In addition, the surface area of the Sb_4_O_5_Cl_2_ samples has also been investigated. The N_2_ adsorption-desorption isotherms of the Sb_4_O_5_Cl_2_ samples prepared at pH = 1, 2, 3, 4 and 5 were shown in Supplementary Fig. S4. The BET surface area of the Sb_4_O_5_Cl_2_ samples prepared at pH = 1, 2, 3, 4 and 5 are calculated to be 14.04, 18.62, 8.69, 8.63, and 8.45 m^2^ g^−1^, respectively. It shows that the samples prepared at pH 3, 4 and 5 have similar BET surface area, which are slightly smaller than the samples prepared at pH 1 and 2. A larger surface area of a photocatalyst is expected to supply more surface active sites, which may lead to an enhanced photocatalytic performance.

## Discussion

The above information clearly shows that the nanoparticles assembled Sb_4_O_5_Cl_2_ crystallites display narrowed bandgap, enhanced photoelectrochemical properties and enhanced BET surface area. With the question whether these features are favorable for solar energy utilization, the photocatalytic performance of the as-prepared Sb_4_O_5_Cl_2_ crystallites was investigated. The photocatalytic activities of as-prepared samples were firstly evaluated by the degradation of gaseous IPA into acetone under ultraviolet light irradiation. Under light irradiation, the photogenerated electrons of photocatalyst can combine with a molecule of O_2_ to produce the •O_2_^−^ radical, and then the •O_2_^−^ oxidizes IPA to become acetone; or the photoexcited holes directly oxidize IPA into acetone^40^,^41^. In this case, the photooxidization of IPA to acetone is a one-photon process. After that, the acetone could be further oxidized into CO_2_ through a multielectron oxidization process^40^,^41^. The clear reaction mechanism and typical intermediate product enable us to evaluate the photocatalytic performance of the samples. Therefore, the photocatalytic degradation of IPA was selected as a model reaction in this work. As shown in Fig. 4(e,f), Sb_4_O_5_Cl_2_ samples prepared at different precursor solution pH values present obviously different acetone and CO_2_ evolution amount (9.00, 10.41, 6.06, 5.64, 5.34 ppm/h for acetone evolution and 2.51, 3.20, 0.60, 0.29, 0.07 ppm/h for CO_2_ evolution, respectively). Sb_4_O_5_Cl_2_ sample prepared at pH = 2 exhibit superior photocatalytic acetone and CO_2_ evolution activity compared with that of other four samples. Control experiments showed that the acetone and CO_2_ produced from IPA in the absence of any catalysts or light irradiation could be neglected, which demonstrated that the acetone and CO_2_ were produced through a photocatalytic pathway. The largest amount of acetone and CO_2_ evolution of Sb_4_O_5_Cl_2_ sample prepared at pH = 2 may be caused by its higher light absorption, the decreased electron-transfer resistance, the suppressed recombination of photogenerated electrons and holes, and the larger surface area which could promote the photooxidization of IPA^42^.

**Figure 5:**
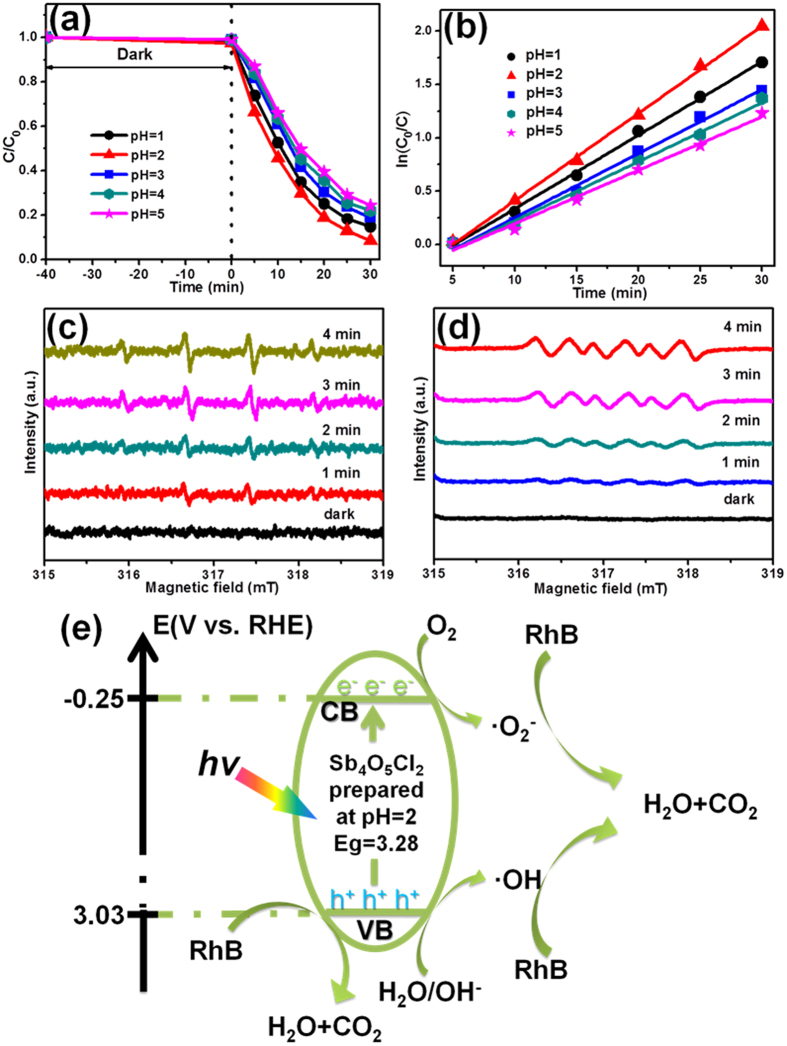
(**a**) Photocatalytic degradation of RhB aqueous solution, and (**b**) the fittings of ln (C_0_/C) plot vs. time over Sb_4_O_5_Cl_2_ samples prepared at pH = 1, 2, 3, 4 and 5, respectively. Electron spin resonance signals of (**c**) DMPO-•OH adducts and (**d**) DMPO-•O_2_^−^ adducts produced by Sb_4_O_5_Cl_2_ sample prepared at pH = 2 before and after light illumination. (**e**) Possible mechanism of the photocatalytic degradation of RhB over the Sb_4_O_5_Cl_2_ sample.

The photocatalytic activities of the as-prepared Sb_4_O_5_Cl_2_ crystallites were also performed by photocatalytic degradation of RhB solution. The photocatalytic reaction was evaluated through monitoring the decolorizating of the UV-vis absorption spectra of RhB solution. The reaction system was stayed in dark for a while to reach adsorption equilibrium before exposed to light irradiation. As shown in Supplementary Fig. S5, all the samples can reach adsorption equilibrium after 40 minutes, and Sb_4_O_5_Cl_2_ crystallites prepared at pH = 2 absorb the largest amount of RhB, due to the largest BET surface area. Figure 5(a) displays RhB solution concentration (C/C_0_) versus the reaction time over Sb_4_O_5_Cl_2_ samples prepared at pH = 1, 2, 3, 4 and 5, respectively. This degradation process of RhB is in accordance with first-order kinetics model of Langmuir-Hinshelwood equation, and its kinetics can be expressed as: ln(C_0_/C) = kt + x, where k is the apparent reaction rate constant, C_0_ is the initial concentration of aqueous RhB, t is the reaction time, and C is the concentration of aqueous RhB at the reaction time of t^43^. And the calculated reaction rate constants (k) were 0.06894, 0.08185, 0.06002, 0.05534 and 0.05007 min^−1^ for x = −0.35323, −0.40657, −0.34718, −0.33038 and −0.30474, respectively, as shown in Fig. 5(b). It is obvious that the Sb_4_O_5_Cl_2_ sample prepared at pH = 2 exhibits superior photocatalytic activity (95% of RhB decomposed after irradiation for 30 min) compared with that of other four samples. The BET surface area, electronic structure and photocatalytic activity of samples prepared at different pH values were summarized in Table 1. It shows that the improved photocatalytic activity of Sb_4_O_5_Cl_2_ sample can be explained as the synergistic effects of larger surface area, higher light absorption, the decreased electron-transfer resistance and the suppressed recombination of photogenerated electrons and holes.

Additionally, to investigate the role of free radical intermediates generated under irradiation in the phtotcatalytic reaction, the hydroxyl radical (•OH) and superoxide radical (•O_2_^−^) were detected by the ESR spin-trapping technique using DMPO (5, 5-dimethyl-1-prroline-*N*-oxide) as spin-trapping agent. Figure 5(c) shows the ESR spectra of the DMPO-•OH over Sb_4_O_5_Cl_2_ sample prepared at pH = 2 in aqueous dispersion before and after irradiation. It should be pointed out that no ESR signals were observed when the measurement was performed in the dark. However, when the reaction system is exposed under irradiation, four characteristic peaks with a peak intensity of 1:2:2:1 of DMPO-•OH adducts were obviously observed^44^. The intensity increased as irradiation time prolongs indicating the more amount of hydroxyl radicals were produced in the reaction system. Figure 5(d) shows the ESR spectra of the DMPO-•O_2_^−^ over Sb_4_O_5_Cl_2_ sample prepared at pH = 2 in methanol dispersion before and after irradiation. The ESR signal is consistent with the characteristic peaks of DMPO-•O_2_^−^ adducts signals^45^. Therefore, the main oxidative species of the reaction system could be •OH and •O_2_^−^ which promote the photocatalytic activity. ESR results confirm that •OH and •O_2_^−^ radicals were produced in the irradiated Sb_4_O_5_Cl_2_ sample. •OH and •O_2_^−^ radicals with strong oxidation capability acted as the predominant active species, which induced the degradation of organic pollutants. Furthermore, it is strongly indicated that the photo-induced electron–holes in the Sb_4_O_5_Cl_2_ sample are long-lived enough to react with adsorbed oxygen and surface hydroxyl groups to produce reactive oxygen species, implying the effective separation of the photo-generated charges and the efficient photocatalytic activity^46^.

To explain the photocatalytic mechanism based on the electronic structures, a schematic illustration for the photocatalytic degradation of RhB over the Sb_4_O_5_Cl_2_ powder was proposed in Fig. 5(e). Under light irradiation the Sb_4_O_5_Cl_2_ photocatalyst is excited and the electrons are promoted from the valence band to its conduction band. The photogenerated electrons are quickly transferred to the catalyst’s surface and then are captured by the absorbed O_2_ to produce •O^2−^, which is responding for the degradation of RhB. At the same time, the holes remained in the Sb_4_O_5_Cl_2_ can react with OH^−^ to produce •OH and subsequently degrade RhB, or take part in the degradation of RhB directly^46^.

It is well-known that the stability and reusability of the photocatalysts are very important from an economic point of view. Supplementary Fig. S6 shows the recyclability of photocatalytic activity of the Sb_4_O_5_Cl_2_ sample prepared at pH = 2 towards the degradation of RhB. It is clear that the Sb_4_O_5_Cl_2_ sample prepared at pH = 2 exhibits stable photocatalytic activity even after three cycles. The above information demonstrates that the as-prepared Sb_4_O_5_Cl_2_ crystallites can be used as stable and efficient photocatalysts.

In conclusion, Sb_4_O_5_Cl_2_ crystallites with photocatalytic performance have been successfully synthesized by a simple hydrothermal route. It is found that the structure, photoelectrochemical and photocatalytic properties of the prepared Sb_4_O_5_Cl_2_ crystallites were significantly dependent on pH values of the precursors. Irregular-cuboids assembled Sb_4_O_5_Cl_2_ micro-particles and hollow microspheres can be synthesized at pH 1 and 2, whereas individual Sb_4_O_5_Cl_2_ micro-belts became to form when the pH is higher than 3. Electronic coupling in these assembled Sb_4_O_5_Cl_2_ crystallites yielded much narrowed bandgaps and red-shifted optical absorption bands compared with individual Sb_4_O_5_Cl_2_ micro-belts. The photoelectrochemical measurements also show that the irregular-cuboids assembled Sb_4_O_5_Cl_2_ crystallites exhibit enhanced carrier density, improved separation efficiency of electron-hole pairs and decreased electron-transfer resistance. As a result, irregular-cuboids assembled Sb_4_O_5_Cl_2_ hollow microspheres prepared at pH = 2 achieved superior aptitude for solar energy utilization. This finding opens up a novel way to narrow the bandgap of Sb_4_O_5_Cl_2_ photocatalyst by electronic coupling in assemblies of nanoparticles rather than by doping foreign elements to promise solar energy utilization. In principle, this electronic coupling assembly of nanoparticles may be extended to other semiconductor materials to tune their electronic structure and band gaps.

## Methods

### Synthesis of Sb_4_O_5_Cl_2_ crystallites

In a typical experimental procedure, 0.01 mol SbCl_3_ was dissolved in 10 mL absolute ethyl alcohol and then slowly added in 40 mL deionized water. Aqua ammonia was dropwise used to adjust the pH value under the continuous magnetic stirring. After that, the precursor was transformed into a Teflon autoclave with a capacity of 100 mL, and then kept at 160 °C for 12 h. After being cooled to room temperature, the white powder was separated by high-speed centrifugation and washed with distilled water for several times. The individual particle sample was prepared by an ultrasound method from the particle-assembled sample. In brief, 0.1 g particle-assembled sample prepared at pH = 2 was dispersed in 100 ml ethanol, and then the mixture was ultrasonicated in a water bath for 12 hours.

### Preparation of Sb_4_O_5_Cl_2_ photoanodes

Clean ITO glass was first obtained by sequentially washing with acetone, distilled water, and ethanol in an ultrasonic cleaner for 30 min. Then, 1.0 mg photocatalyst was well dispersed in alcohol containing nafion and stayed in an ultrasonic cleaner for 30 min. Subsequently, the suspension was doped onto 1 × 2 cm^2^ ITO glass uniformly. The boundary of ITO glass was protected using Scotch tape. After drying overnight in an oven, the electrodes were sintered at 200 °C for 2 h to improve adhesion. The loading mass is about 1 mg/cm^2^.

### Characterization

The crystalline structures of the samples were characterized by an X-ray diffractometer (XRD, D/max-2200, Rigaku) with Cu Kα radiation (λ = 0.15418 nm). A field emission scanning electron microscope (FE-SEM, JSM-6700F, JEOL) was used to examine the morphology and the particle size of the samples. TEM, HRTEM and TEM elemental mapping images were taken on a transmission electron microscope (TEM, 2100F, JEOL Co., Japan). X-ray photoelectron spectroscopy (XPS, PHI Quantera SXM, ULVAC-PHI Inc., Japan) was used to analyze the compositions and chemical state of the samples. UV–vis diffuse reflectance spectra were obtained on an UV–vis spectrophotometer (Hitachi U-3900H) in the wavelength range of 200–800 nm, using BaSO_4_ as a reference. The Brunauer–Emmett–Teller (BET) surface area was measured with a 3H-2000 BET-A surface area analyzer. Mott-Schottky plots, photocurrent response, and electrochemistry impedance spectroscopy (EIS) measurements were performed with a CHI electrochemical analyser (ALS/CH model 650A) using a standard three-electrode mode with 0.5 M Na_2_SO_4_ (pH 6.8) solution as the electrolyte, Ag/AgCl (saturated KCl) as the reference electrode and a Pt sheet as the counter electrode. The cleaned indium tin oxide (ITO) deposited with Sb_4_O_5_Cl_2_ samples prepared at pH = 1, 2, 3, 4 and 5 through hydrothermal process (T = 160 °C, t = 12 h) were fabricated respectively as photoanodes. The simulated sunlight was obtained using an AM 1.5 solar simulator (WXS-80C-3 AM 1.5G) with a light intensity of 0.1 W/cm^2^. Mott-Schottky plots were collected at 1000 Hz and 1500 Hz respectively in the dark. The photocurrent response of the photocatalysts as light on and off was measured with 0.5 V bias voltage. EIS measurements were carried out at zero potential in the frequency range of 1 Hz to 100 kHz with 10 mV amplitude. Electron paramagnetic resonance (ESR) signals of radicals species with 5,5-dimethyl-1-pyrroline-N-oxide (DMPO) were recorded at room temperature with a JEOL ESR JES-FA200 spectrometer. The spectral parameters for the ESR are shown in below: centre field = 321 mT, sweep width = 7.5 mT, microwave frequency = 8.99 GHz, modulation frequency = 100 KHz and power = 2 mW.

### Photocatalytic activities

To evaluate the photocatalytic activities of as-synthesized samples, the photodegradation of gaseous iso-propanol (IPA) and the photodegradation of Rhodamine B (RhB) aqueous solution were chosen as the probe reactions. For the photocatalytic degradation of IPA, a 300-W Xe arc lamp was employed as the light source of photocatalytic reaction. The reactor volume was 500 mL and it was equipped with a Pyrex-glass lid as a window. 200 mg of Sb_4_O_5_Cl_2_ sample was spread uniformly on an 8.5 cm^2^ plate which was placed in the bottom of the reactor. Then the reactor was pretreated by artificial air [V_(N2)_: V_(O2)_ = 4:1] for 10 min to remove adsorbed gaseous impurities. A certain amount of the gaseous IPA was injected into the reactor. Before irradiation, the reactor was kept in the dark until ensuring an adsorption-desorption equilibrium of gaseous reactants on the sample. The concentrations of acetone and CO_2_ were detected on a gas chromatograph (GC-14B; Shimadzu Corp., Japan) with an FID detector (Details: Porapak Q and PEG1000 column; temperatures-injection port, 120 °C; column, 60 °C; detection, 200 °C; maximum error about 7%). The IPA photodegradation undergoes two kinds of typical reaction processes as below^40^,^41^:

one-photon reaction:


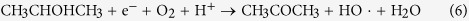



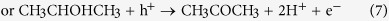


multiphoton reaction:


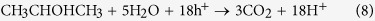


The photodegradation of Rhodamine B (RhB) experiments were carried out as follows: 50 mg of the photocatalyst was added to 50 mL RhB solution (10 mg/L). Before the irradiation, the mixed solution was magnetically stirred for 40 min in the dark to ensure the establishment of the adsorption–desorption equilibrium. Then, the suspension was exposed to the light irradiation under magnetically stirring. A 300-W Xe arc lamp was used as the light source of photocatalytic reaction. The concentration of RhB was determined on a UV–vis spectrophotometer (Unico, UV-2600) by monitoring its characteristic absorption at 558 nm.

## Additional Information

**How to cite this article**: Yang, L. *et al*. pH-regulated template-free assembly of Sb_4_O_5_Cl_2_ hollow microsphere crystallites with self-narrowed bandgap and optimized photocatalytic performance. *Sci. Rep*. **6**, 27765; doi: 10.1038/srep27765 (2016).

## Supplementary Material

Supplementary Information

## Figures and Tables

**Table 1:** Structural, morphology and photocatalytic activity information of the as-synthesized Sb_4_O_5_Cl_2_ crystallites synthesized by varying the pH values.

PrecursorpH values	Morphologies	BET(m^2^ g-1)	Band gapEg (eV)	VB position(eV)	CB position(eV)	Acetone evolution(ppm/h)	CO_2_ evolution(ppm/h)	RhB degradationreaction rate constant (k)
pH = 1	Particles assembled byirregular cuboids	14.04	3.29	−0.20	3.09	9.00	2.51	0.06894
pH = 2	hollow microspheresassembled by irregular cuboids	18.62	3.28	−0.25	3.03	10.41	3.20	0.08185
pH = 3	belts	8.69	3.36	−0.18	3.18	6.06	0.60	0.06002
pH = 4	belts	8.63	3.37	−0.13	3.24	5.64	0.29	0.05534
pH = 5	belts	8.45	3.38	−0.10	3.28	5.34	0.07	0.05007
