# Supplementary material for: pH-regulated template-free assembly of Sb4O5Cl2 hollow microsphere crystallites with self-narrowed bandgap and optimized photocatalytic performance
Source: Sci Rep. 2016 Jun 16;6:27765. doi: 10.1038/srep27765 (PMC4910059; doi:10.1038/srep27765)
Supplement: Supplementary Information [file srep27765-s1.pdf]

Supplementary Information

pH-regulated template-free assembly of $Sb_4O_5Cl_2$ hollow microsphere crystallites with self-narrowed bandgap and optimized photocatalytic performance

Liuqing Yang[1], Jianfeng Huang[1,*], Liyun Cao[1,*], Li Shi[2], Qing Yu[2], Xingang Kong[1], Yanni Jie[1]

1 School of Materials Science and Engineering, Shaanxi University of Science and Technology, Weiyang, Xi'an, Shaanxi 710021, PR China.

2 Environmental Remediation Materials Unit, National Institute for Materials Science (NIMS), 1-1 Namiki, Tsukuba, Ibaraki 305-0044, Japan.

[*]E-mail adress: huangjf@sust.edu.cn; caoliyun@sust.edu.cn

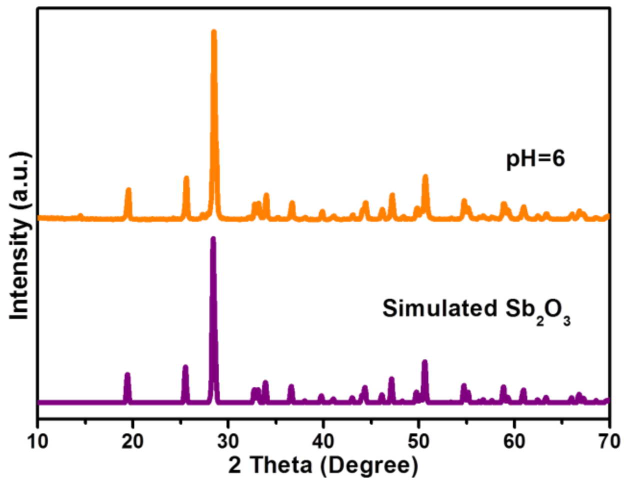

Supplementary Fig. S1. XRD patterns of the samples prepared at precursor pH= 6 through hydrothermal process (T=160 °C, t=12 h).

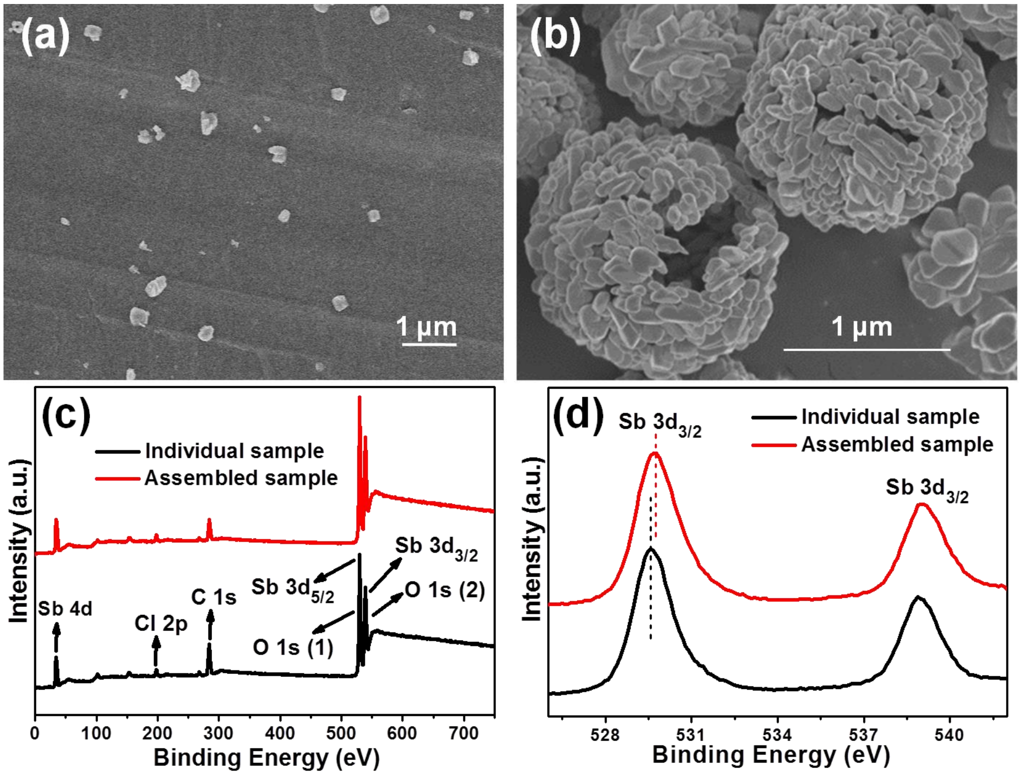

Supplementary Fig. S2. SEM images of (a) $Sb_4O_5Cl_2$ individual particles sample obtained from the ultrasound treatment of $Sb_4O_5Cl_2$ particle-assembled sample prepared at pH=2 and (b) $Sb_4O_5Cl_2$ particle-assembled sample prepared at pH=2. (c) XPS spectra of the corresponding individual particles sample and particle-assembled sample. (d) High resolution Sb 3d spectra of individual particles sample and particle-assembled sample.

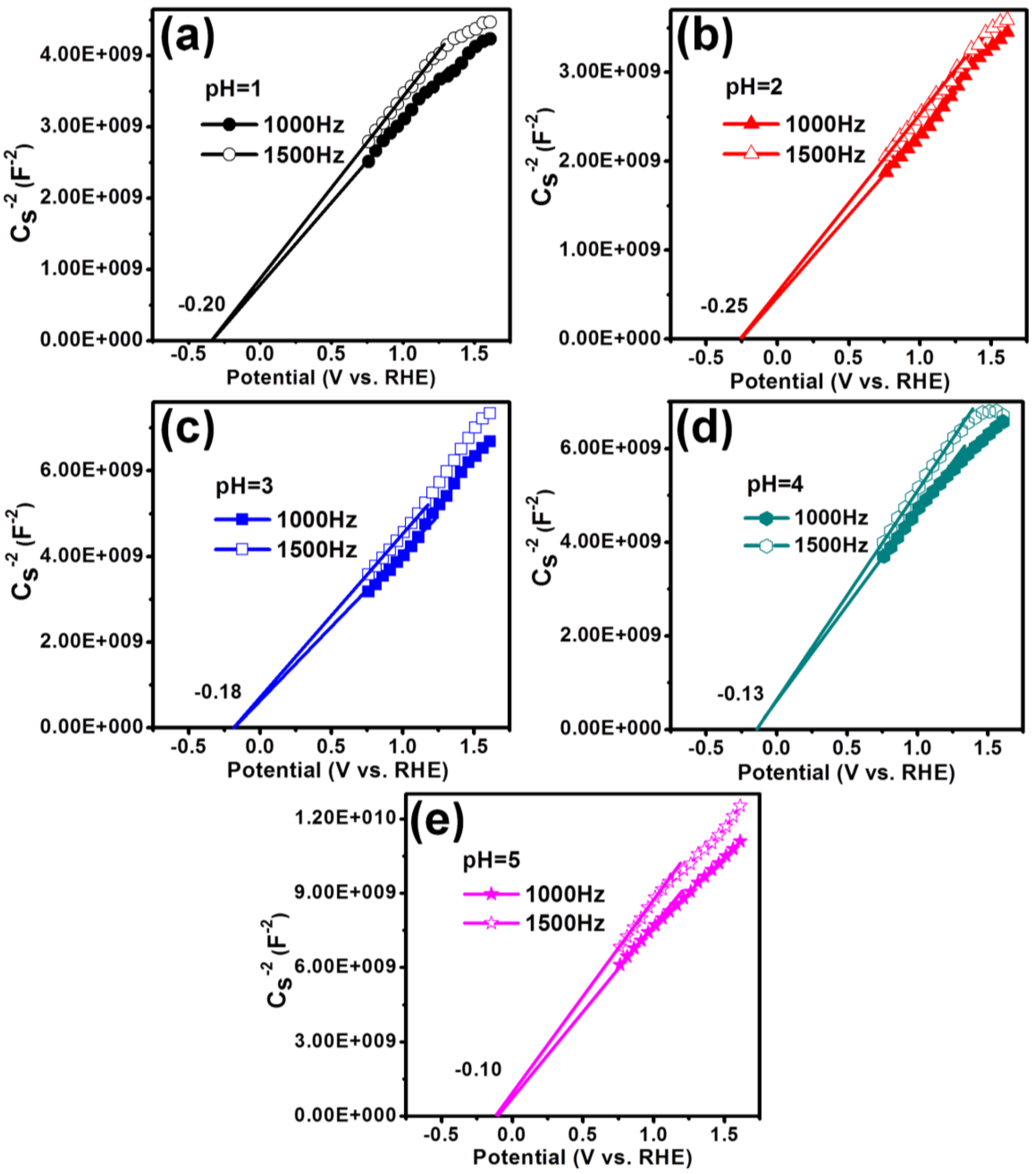

Supplementary Fig. S3. Mott-Schottky curves of $Sb_4O_5Cl_2$ crystals prepared (a) pH=1, (b) 2, (c) 3, (d) 4 and (e) 5 at the select frequencies of 1000 and 1500 Hz in 0.5 M Na_2SO_4 aqueous solution.

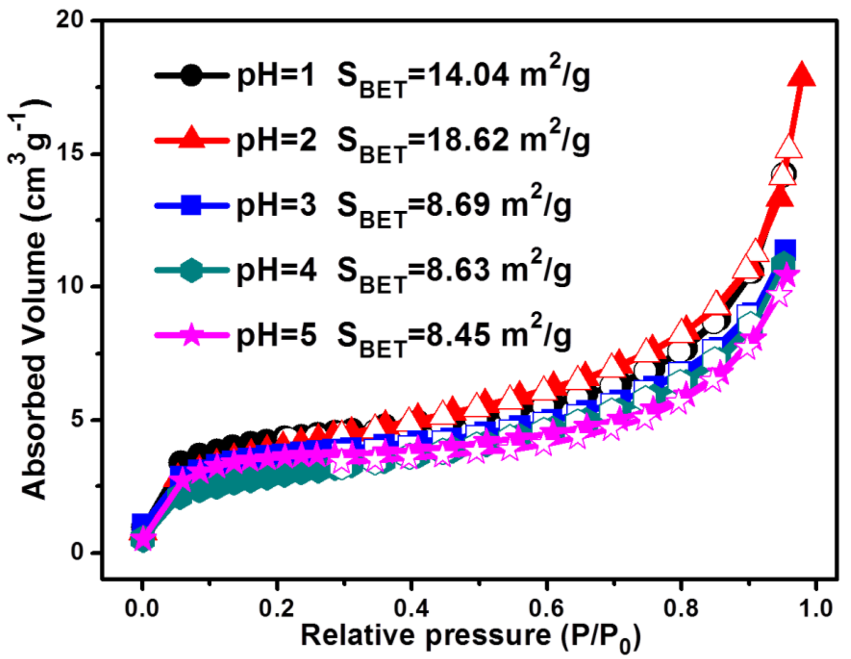

Supplementary Fig. S4. BET adsorption-desorption isotherms of the $Sb_4O_5Cl_2$ samples prepared at pH=1, 2, 3, 4 and 5 through hydrothermal process (T=160 °C, t=12h).

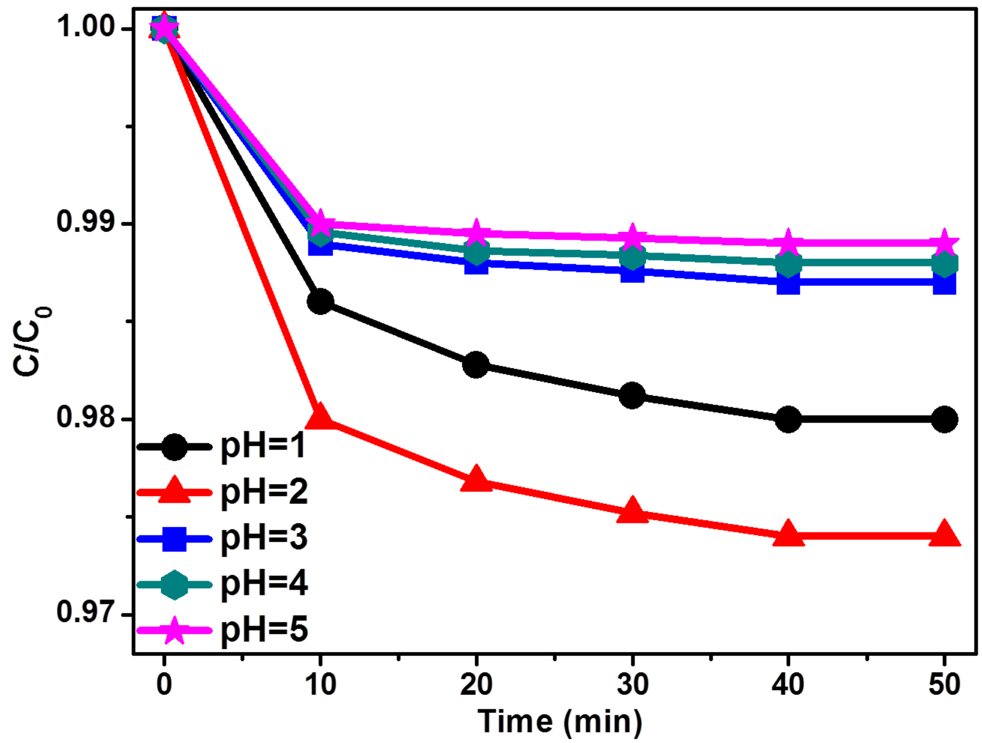

Supplementary Fig. S5. Adsorption curves (C_0/C plots vs. time) of 10 mg/mL RhB aqueous solution over $Sb_4O_5Cl_2$ samples prepared at pH=1, 2, 3, 4 and 5, respectively. The experiments were conducted in the dark.

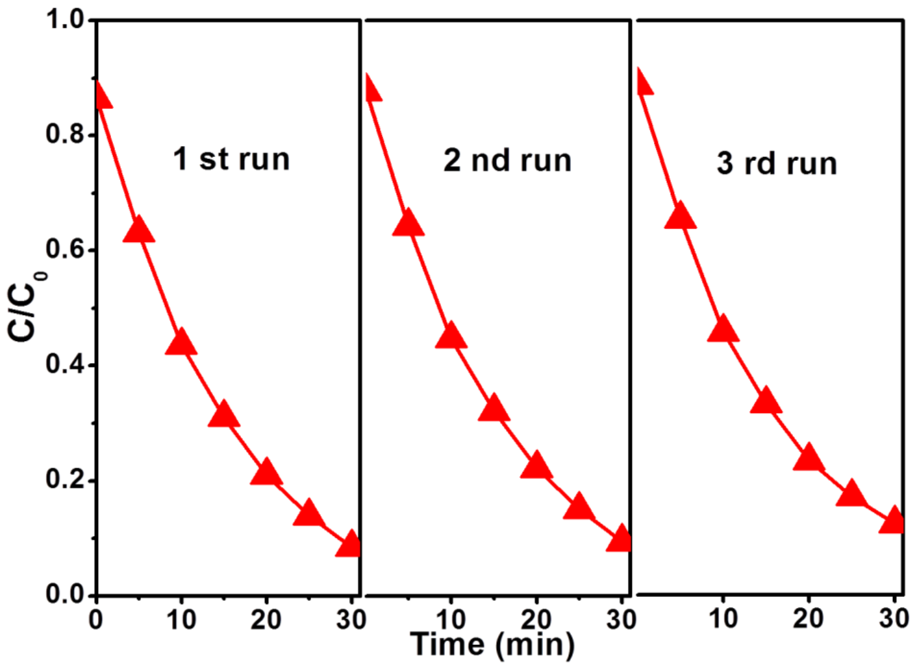

Supplementary Fig. S6. Photocatalytic cycling degradation for 10 mg/mL RhB aqueous solution with $Sb_4O_5Cl_2$ sample prepared at pH=2 under illumination of a 300 W Xe lamp.

Supplementary Tab. S1. Performance comparison of $Sb_4O_5Cl_2$-based materials as photocatalyst for organic dyes degradation.

Photocatalyst (amount)	Light source	Reaction solution	Time (min)	Degradation efficiency (%)	Reference
$Sb_4O_5Cl_2$ (50 mg)	300 W Xe lamp	50 mL of 10 mg/L RhB solution	30	95	In this work
$Sb_2S_3/Sb_4O_5Cl_2$ composite photocatalysts (100 mg)	500 W Xe lamp irradiation with a 420 nm cut-off filter	100 mL of 15 mg/L MO solution	60	82.9	1
g-C_3N_4–$Sb_2S_3/Sb_4O_5Cl_2$ composite photocatalysts (100 mg)	500 W Xe lamp irradiation with a 420 nm cut-off filter	100 mL of 20 mg/L MO solution	60	95	2

1. Jiang, Q. et al. A facile hydrothermal method to synthesize $Sb_2S_3/Sb_4O_5Cl_2$ composites with three-dimensional spherical structures. *RSC Adv.* **5**, 53019-53024 (2015).
2. Liu, Y. et al. Novel visible light-induced g-C_3N_4–$Sb_2S_3/Sb_4O_5Cl_2$ composite photocatalysts for efficient degradation of methyl orange. *Catal. Commun.* **70**, 17-20 (2015).
